# Comparison of Volatile and Nonvolatile Compounds in Rice Fermented by Different Lactic Acid Bacteria

**DOI:** 10.3390/molecules24061183

**Published:** 2019-03-26

**Authors:** Sang Mi Lee, Young Rim Hwang, Moon Seok Kim, Myung Sub Chung, Young-Suk Kim

**Affiliations:** 1 Department of Food Science and Engineering, Ewha Womans University, Seoul 120-750, Korea; smlee78@ewha.ac.kr (S.M.L.); hhyr9@naver.com (Y.R.H.); 2 Sempio Foods Company R&D Center, Cheongju 363-954, Korea; kmoonseok@sempio.com; 3 Department of Food Science and Technology, Chung Ang University, 4726 Seodongdae-ro, Daeduk-myun, Ansung, Gyungki-do 17546, Korea; chungms@cau.ac.kr

**Keywords:** lactic acid bacteria, volatile compounds, nonvolatile compounds, fermented rice

## Abstract

The production of rice-based beverages fermented by lactic acid bacteria (LAB) can increase the consumption of rice in the form of a dairy replacement. This study investigated volatile and nonvolatile components in rice fermented by 12 different LABs. Volatile compounds of fermented rice samples were analyzed using gas chromatography-mass spectrometry (GC-MS) combined with solid-phase microextraction (SPME), while nonvolatile compounds were determined using gas chromatography-time-of-flight/mass spectrometry (GC-TOF/MS) after derivatization. The 47 identified volatile compounds included acids, aldehydes, esters, furan derivatives, ketones, alcohols, benzene and benzene derivatives, hydrocarbons, and terpenes, while the 37 identified nonvolatile components included amino acids, organic acids, and carbohydrates. The profiles of volatile and nonvolatile components generally differed significantly between obligatorily homofermentative/facultatively heterofermentative LAB and obligatorily heterofermentative LAB. The rice sample fermented by *Lactobacillus sakei* (RTCL16) was clearly differentiated from the other samples on principal component analysis (PCA) plots. The results of PCA revealed that the rice samples fermented by LABs could be distinguished according to microbial strains.

## 1. Introduction

The grains such as rice, wheat, oats, malt, and barley are widely used as ingredients for improving the functional properties in diverse foods [1,2]. In particular, grain-based probiotic functional foods are becoming increasingly popular and have considerable potential as dairy replacements [2,3].

Grains can be used as substrates to produce fermented foods by lactic acid bacteria (LAB) [4,5]. Several studies have demonstrated the fermentation of LAB in grain substrates [4,5,6,7,8]. Marklinder and Johansson found that grain-based nondairy products were suitable for the growth of probiotic LAB strains for fermentation [6]. Angelov et al. utilized whole grains as a substrate for fermenting LAB to produce a grain-based beverage [4]. The role of LAB in rice fermentation has also been reported [8,9,10,11]. Steamed breads such as *idli* and *puto*, which are made with rice, are fermented by *Leuconostoc* strains, with *Leuconostoc mesenteroides* in particular initiating relatively rapid growth in rice [8,9]. *Chicha* and *Haria*, which are rice-based ethnic fermented beverages of east and central India and Brazil, are fermented simultaneously by LAB and yeast [10,11].

Fermentation by LAB can change the taste and aroma of substrates and has been used to improve the organoleptic quality of fermented foods [2]. LAB can produce large amounts of lactic acid via diverse metabolic pathways and form volatile compounds derived from amino acids, peptides, and fatty acids upon further bioconversions [12,13]. Some studies have reported the formation of volatile compounds in grain substrates and grain-based products by LAB [1,14,15]. Salmerón et al. demonstrated the formation of volatile compounds by *Lactobacillus plantarum* NCIMB 8826 (NCIMB 8826; National Collection of Industrial and Marine Bacteria, UK) grown in grain substrates (wheat, oats, malt, and barley) [14]. Those authors identified 60 compounds, with the most abundant in all grain substrates (wheat, oats, malt, and barley) being linoleic acid, oleic acid, 5-hydroxymethylfurfural, and acetic acid, respectively [14]. In addition, Salmerón et al. employed headspace gas chromatography analysis to determine volatile compounds formed from barley and malt fermented by *Lactobacillus reuteri*, *Lac. plantarum*, and *Lactobacillus acidophilus* [1]. They found that these *Lactobacillus* strains can produce large amounts of certain major flavor compounds (acetaldehyde, ethanol, acetone, diacetyl, and ethyl acetate), which affects the organoleptic qualities of the fermented products [1]. In particular, fermentation by LAB generates characteristic aroma compounds such as acetaldehyde, acetone, butan-2-one, butane-2,3-dione (diacetyl), and 3-hydroxybutan-2-one (acetoin) in fermented products [15]. Butane-2,3-dione and 3-hydroxybutan-2-one, which have creamy and buttery aroma notes, can serve as major flavor compounds in various dairy products fermented by LAB [13,16,17].

LAB can also contribute to the formation of nonvolatile compounds that can act as precursors of characteristic aroma compounds as well as tastants themselves in fermented products such as ripened cheese, sourdough, butter, buttermilk, fermented vegetables, and yogurt [18]. Drinan et al. investigated the effects of citrate on the formation of butane-2,3-dione and 3-hydroxybutan-2-one by different LAB strains [19]. The heterofermentative LAB did not produce butane-2,3-dione or 3-hydroxybutan-2-one in the absence of citrate, whereas all of the homofermentative LAB produced significant amounts of butane-2,3-dione and 3-hydroxybutan-2-one in the presence of citrate. In addition, lactones can be formed from unsaturated fatty acids such as oleic acid and linoleic acid during fermentation [13,20]. It has been reported that these lactones can contribute significantly to the flavor characteristics of fermented rice foods such as rice beer and Chinese rice wine [13,21,22].

Rice is widely consumed as the staple food in many Asian countries and has considerable potential as a dairy replacement via the application of fermentation. However, no study has compared the formation of volatile and nonvolatile compounds in rice fermented by various LABs, which have different metabolic activities. Accordingly, the present study investigated and compared the formation of volatile and nonvolatile compounds in rice fermented by 12 different LABs.

## 2. Results and Discussion

### 2.1. Volatile Compounds in Fermented Rice Samples

The volatile compounds in the fermented rice samples inoculated by LAB are listed in Table 1. The 45 identified volatile compounds were comprised of three acids, five aldehydes, four esters, four furan derivatives, four ketones, 13 alcohols, 10 benzene and benzene derivatives, one hydrocarbon, and one terpene. 

Esters, which have floral and fruity odor notes, are formed by esterification between alcohols and acids during LAB metabolism [23]. The present study detected four esters (butyl acetate, ethyl acetate, hexyl acetate, and methyl butanoate) in the fermented rice samples, although they were present at only low levels. In particular, ethyl acetate and methyl butanoate were found in all of the samples studied. These compounds, which were formed from pyruvate metabolism, were previously identified as key aroma compounds in fermentation products by *Lactobacillus* [13]. On the other hand, butyl acetate was detected in only five samples (KR7, KR10, JKA1-6, JFK2-2, and RTCL9), whereas hexyl acetate was observed only in samples KR7 and KR10; both of these LABs are *Lactobacillus brevis* strains. Esters were generally most abundant in JKA1-6, with the largest amounts in samples fermented by obligatorily heterofermentative LAB except for sample RTCL16. It was considered that heterofermentative LABs produce more ethanol and acetic acid [19], which can be better used for esterification compared to facultatively hetero-/homo- ones.

Various alcohols and acids are produced via the pentose phosphate pathway and the TCA cycle of LAB [24]. Ethanol was the predominant alcohol in most of the fermented rice samples in the present study, and is mainly formed from alcoholic fermentation. In homofermentative LAB, ethanol is derived from pyruvate via acetyl-CoA, whereas it is derived from the pentose phosphate pathway of glucose in heterofermentative LAB [24]. The level of ethanol was much higher in samples KR10 and KR7, which are *Lac. brevis* strains. Annuk et al. reported that *Lac. brevis* produced more ethanol than other LABs [25]. In addition, the level of ethanol differed significantly between obligatorily heterofermentative and facultatively heterofermentative/obligatorily homofermentative LAB samples in the present study.

1-Octen-3-ol, which can be produced from unsaturated fatty acids with some microbial involvement via an enzymatic reaction [26], was found in samples RTCL16, JFK2-2, JKA1-6, and RTCL9. Matsui et al. reported that 1-octen-3-ol could be formed from 10-hydroperoxide of linoleic acid [26]. Linoleic acid would be hydroperoxided by lipoxygenase, producing 10-hydroperoxylinoleic acid, and then hydroperoxide lyase would decompose 10-hydroperoxylinoleic acid into 1-octen-3-ol and 10-oxo-trans-8-decenoic acid [26,27].

(*E*)-Hept-2-en-1-ol and heptan-2-ol were observed in only obligatorily heterofermentative LAB and not in facultatively heterofermentative or obligatorily homofermentative LAB. 3-Methylbutan-l-ol can be formed by the reduction of 3-methylbutanal produced by the Strecker degradation of leucine, and RTCL16 contained the most 3-methylbutan-l-ol. Vermeulen et al. reported that *Lactobacillus sanfranciscensis*, which is classified as heterofermentative, reduced aldehydes to the corresponding unsaturated alcohols, whereas *Lactobacillus sakei*, which is facultatively heterofermentative, did not metabolize the unsaturated aldehydes at all [28]. In heterofermentative LAB, the reduction of aldehydes is related to the oxidation of NADH to NAD+, which enables this heterofermentative LAB to produce additional ATP from glucose [28]. However, LAB, which metabolize glucose via the Embden-Meyerhof pathway, were not affected from NADH recycling during growth in sourdough [28].

2-Pentylfuran, which has a beany odor note, has been reported as one of the odor-active compounds in various rice cultivars [29] and is mainly derived from the specific oxidation of 9-hydroperoxides by lipoxygenase. It could be produced at high levels during fermentation, possibly due to the large amount of linoleic acid and the high lipoxygenase activity in grains [30]. In addition, furan derivatives could have been generated by thermal degradation during sample preparation in the present study. In particular, sample RTCL16 contained the largest amounts of furans, whereas the amounts of furans did not differ significantly between the other samples.

The amounts of aldehydes such as 3-methylbutanal, hexanal, and nonanal were larger than those of heterofermentative LAB. Kaseleht et al. compared the formation of volatile compounds by LAB during the fermentation of rye sourdough with an uninoculated rye sourdough control sample [31]. They reported that aldehydes were more strongly represented in homofermentative than heterofermentative LAB, probably due to the higher alcohol dehydrogenase activity and/or the higher surplus of reductive power (NADH) during the growth of heterofermentative LAB.

Hexanal, which contributes a fatty-green odor note, can be readily recognized by its low odor threshold (5 ng/g) in rice [32]. Hexanal mainly derives from the oxygenation of linoleic acid via the sequential actions of lipoxygenase and hydroperoxide lyase [33]. Hexanal is related to further reactions such as the oxidation to hexanoic acid by aldehyde dehydrogenase and the reduction to 1-hexanol by alcohol dehydrogenase during fermentation [34]. Hexan-1-ol was much more abundant than hexanal and hexanoic acid in all of the samples in the present study. The combined amount of hexanal, 1-hexanol, and hexanoic acid was highest in sample RTCL16, followed by sample RTCL3.

Nonanal, which can be generated by the decomposition of 9-hydroperoxy-octadecadienoic acid derived from the oxidation of oleic acid [2], was detected only in samples RTCL79 and RTCL31. The amount of its corresponding alcohol, nonan-1-ol, was larger than that of nonanal, as was the case for hexanal and hexan-1-ol. Czerny and Schieberle (2002) found that the concentrations of (*E*)-2-nonenal were significantly decreased during sourdough fermentation [35]. The activity of short-chain alcohol dehydrogenase of LAB contributes to the reduction of these flavor compounds [35]. 

2-Methylbutanal and 3-methylbutanal can be formed enzymatically or nonenzymatically by the Strecker degradation of isoleucine and leucine, respectively. Only two samples in this study (RTCL3 and RTCL16) produced both 2-methylbutanal and 3-methylbutanal. RTCL31 contained only 2-methylbutanal, whereas 3-methylbutanal was only detected in RTJL3 and LPC. Leucine produces 3-methylbutanal by Strecker degradation, and this is subsequently reduced to 3-methyl-1-butanol by alcohol dehydrogenase. 3-Methyl-1-butanol was detected in all of the samples, whereas 3-methylbutanal was found only in three samples—RTJL3, LPC, and RTCL3. 

The ketones detected in fermented rice were methyl ketones including propan-2-one, butan-2-one, 6-methylhept-5-en-2-one, and nonan-2-one. Methyl ketones, which have a characteristic aroma note of ripened cheeses, are formed from fatty acids by enzymatic oxidative decarboxylation (β-oxidation) [2]. Ketones were not detected in sample RTJL4 fermented by *Lactobacillus hilgardii*, while the amount of propan-2-one was largest in sample RTCL16.

A particularly interesting finding of the present study was that the amounts of 3-hydroxybutan-2-one and butane-2,3-dione were much larger for the homofermentative/facultatively heterofermentative LAB RTJL3, LPC, RTCL16, RTCL79, and RTCL31 than for the obligatorily heterofermentative LAB KR10, KR7, RTJL4, JFK2-2, JKA1-6, and RTCL9. 3-Hydroxybutan-2-one and butane-2,3-dione can be produced by the metabolism of LAB by glucose, citrate, and aspartic acid [19]. Drinan et al. found that *Lac. plantarum* and *Streptococcus lactis*, which were classified as homofermentative LAB, could produce larger amounts of butane-2,3-dione and 3-hydroxybutan-2-one than could *Lactobacillus viridescens*, *Lactobacillus fermenti*, and *Leuconostoc spp*., which are included in heterofermentative LAB [19]. It was also reported that heterofermentative LAB did not produce butane-2,3-dione or 3-hydroxybutan-2-one [19]. Those authors considered that citrate was utilized in some other metabolic pathway during growth in heterofermentative LAB. The largest amounts of butane-2,3-dione and 3-hydroxybutan-2-one were found in RTJL3, followed by LPC, which are both *Lactobacillus paracasei* species classified as facultatively heterofermentative LAB. RTJL3 and LPC produced much lower levels of ethanol than did the obligatorily heterofermentative LAB KR10, KR7, RTJL4, JFK2-2, JKA1-6, and RTCL9, and similar levels to those of the homofermentative LAB RTCL3 and RTCL31. It was considered that low-ethanol-tolerant microorganisms could preferentially switch pyruvate utilization to an acetoin biosynthetic pathway in order to dispose of ethanol that was present at toxic levels [36]. In addition, relatively large amounts of lactic acid were observed in RTJL3 and LPC, while these two samples had low levels of acetic acid. These results are consistent with Pruckler et al. reporting that *Lac. plantarum* and *Lactobacillus pentosus*, which are facultatively heterofermentative LAB, produced a large amount of lactic acid but little acetic acid, exhibiting a profile close to homofermentative fermentation [37]. 

### 2.2. Nonvolatile Compounds in Fermented Rice Samples

The 37 identified nonvolatile compounds with their relative peak areas are listed in Table 2. They were comprised of 17 amino acids, seven organic acids, and 13 carbohydrates. Amino acids were most abundant in KR10 and least abundant in RTCL79. In particular, the amounts of phenylalanine and branched-chain amino acids such as valine, leucine, and isoleucine were much smaller in RTCL79 than in the other samples. Valine, isoleucine, leucine, and phenylalanine can be transformed into Strecker aldehydes, leading to 2-methylpropanal, 2-methylbutanal, 3-methylbutanal, and phenylacetaldehyde, respectively [38]. These Strecker aldehydes can form the alcohols 2-methylpropan-1-ol, 2-methylbutan-l-ol, 3-methylbutan-l-ol, and phenylethanol [38]. Phenylacetaldehyde is also the most effective precursor for the production of benzaldehyde [39]. The present study identified the presence of 2-methylbutanal, 3-methylbutanal, 3-methylbutan-l-ol, and benzaldehyde. 

Glutamic acid, which contributes to a savory and umami taste, was not detected in RTCL79 or RTJL4. Glutamic acid can be converted into γ-aminobutyric acid (GABA) by glutamate decarboxylase to regulate the internal pH in an acid environment [40]. GABA, which is a nonprotein amino acid, plays an important role in the sympathetic nervous system and cardiovascular function [41].

Ornithine was found in only six samples (RTCL3, RTCL31, KR7, KR10, RTCL16, and RTJL4), which constitute two *Pediococcus sp*., two *Lac. brevis sp*., *Lac. sakei sp*., and *Lac. hilgardii sp*. Ornithine is a central component of the urea cycle that facilitates the disposal of excess nitrogen and can be produced from arginine via the arginine deiminase pathway [42]. Many heterofermentative LABs have the ability to produce energy by utilizing arginine in the formation of ornithine, NH_3_, CO_2_, and ATP [43]. The conversion of arginine via citrulline into ornithine releases ammonia, which increases the pH of the medium and improves the survival of bacteria under acid stress conditions [43]. Previous studies have found that this is not only the case in obligatorily heterofermentative LAB such as *Lac. sanfranciscensis* and some strains of *Lactobacillus buchneri*, *Lac. hilgardii*, *Lac. reuteri*, and *Oenococcus oeni*, but also in the facultative heterofermentative *Lac. plantarum* [43].

Lactic acid derived from pyruvate by lactate dehydrogenase was the predominant organic acid in the present study. Lactic acid not only improves the organoleptic properties of fermented foods [44] but also inhibits the growth of spoilage bacteria in food products. In the present study, lactic acid was much more abundant in KR7 than in rice samples fermented by other LAB. It was considered that KR7 can produce the largest amount of lactate dehydrogenase. 

Citric acid, succinic acid, fumaric acid, and malic acid play important roles in the TCA cycle. The metabolism of citric acid is initiated by citrate permease or citrate lyase. Citrate permease leads to the formation of succinic acid, whereas citrate lyase results in decarboxylation to pyruvate that can be converted into α-acetolactate, which in turn is enzymatically reduced to 3-hydroxybutan-2-one or nonenzymatically transformed into butane-2,3-dione [45]. However, the citrate conversion to succinic acid appears to be more common in *Lactobacillus* strains [16,45]. The amounts of succinic acid, fumaric acid, malic acid, and citric acid were largest in KR7, JKA1-6, RTJL4, and JFK2-2, respectively, whereas there were only small amounts of 3-hydroxybutan-2-one and butane-2,3-dione in these four samples. LABs have a strong tendency to generate organic acids involved in the TCA cycle but rarely appear to produce 3-hydroxybutan-2-one and butane-2,3-dione [16].

Carbohydrates are used as carbon sources to provide microbial energy for the growth of microorganisms via carbohydrate metabolic pathways [2]. Mannitol, which is included in sugar alcohol, can serve as an antioxidant and sweetener in foods [46]. Mannitol was detected in all of the obligatorily heterofermentative LAB except *Weissella cibaria* in the present study, while it was not found in any of the facultatively heterofermentative/homofermentative LAB samples. In particular, JKA2-2 and JKA1-6, which are fermented by *Leuconostoc mesenteroides*, contained large amounts of mannitol. Wisselink et al. demonstrated that several heterofermentative LABs produce large amounts of mannitol using fructose as an electron acceptor, whereas homofermentative LABs only produce small amounts of mannitol [46]. It was considered that two different key enzymes are involved in mannitol production: (1) mannitol 1-phosphate dehydrogenase for homofermentative LAB and (2) mannitol dehydrogenase for heterofermentative LAB. Salminen et al. reported that many heterofermentative LAB gain additional energy by converting acetyl phosphate into acetate instead of ethanol [45]. 

### 2.3. Principal Component Analysis of Fermented Rice Samples according to Strains of LABs

**Figure 1:**
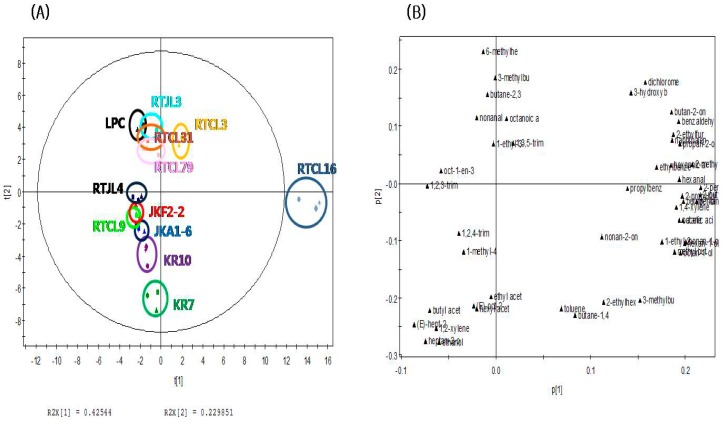
Principal component analysis (PCA) plots of the volatile compounds identified in fermented rice according to strains of LABs: (**A**) score plot, (**B**) loading plot.

The present study applied principal component analysis (PCA) to compare the differences of volatile and nonvolatile compounds among the fermented rice samples according to strains of LABs. The PCA score plot of volatile compounds in Figure 1A shows that rice samples fermented by *Lac. sakei* could be distinguished from the others along the PC1 dimension (explaining 42.5% of the variance). In addition, rice samples fermented by obligatorily homofermentative/facultatively heterofermentative LABs, such as LPC, RTJL3, RTCL16, RTCL79, RTCL3, and RTCL31, were separated from fermented rice samples inoculated with obligatorily heterofermentative lactic acid bacteria, such as KR10, KR7, RTJL4, JKA1-6, JKF2-2, and RTCL9, along the PC2 dimension (explaining 23.0% of the variance). The PCA loading plot of volatile compounds in Figure 1B shows that 2-ethylfuran, 2-propylfuran, 2-butylfuran, 2-pentylfuran, hexanal, 2-methylbutanal, pentan-1-ol, and butan-1-ol were closely correlated with RTCL16. On the other hand, ketones and aldehydes, such as butane-2,3-dione, 3-hydroxybutan-2-one, 6-methylhept-5-en-2-one, 2-methylbutanal, and nonanal, were related to the positive axis of the PC2 dimension. PCA analysis was performed to compare the differences among the fermented rice samples according to strains of LABs. The PCA score plot of volatile compounds in Figure 1A shows that rice samples fermented by LAB inoculated with *Lac. sakei* could be distinguished from the others along the PC1 dimension (explaining 42.5% of the variance). In addition, rice samples fermented by obligatorily homofermentative/facultatively heterofermentative lactic acid bacteria, such as LPC, RTJL3, RTCL16, RTCL79, RTCL3, and RTCL31, were separated from fermented rice samples inoculated with obligatorily heterofermentative lactic acid bacteria, such as KR10, KR7, RTJL4, JKA1-6, JKF2-2, and RTCL9, along the PC2 dimension (explaining 23.0% of the variance). The PCA loading plot of volatile compounds in Figure 1B shows that 2-ethylfuran, 2-propylfuran, 2-butylfuran, 2-pentylfuran, hexanal, 2-methylbutanal, pentan-1-ol, and butan-1-ol were closely correlated with RTCL16. On the other hand, ketones and aldehydes, such as butane-2,3-dione, 3-hydroxybutan-2-one, 6-methylhept-5-en-2-one, 2-methylbutanal, and nonanal, were related to the positive axis of the PC2 dimension.

**Figure 2:**
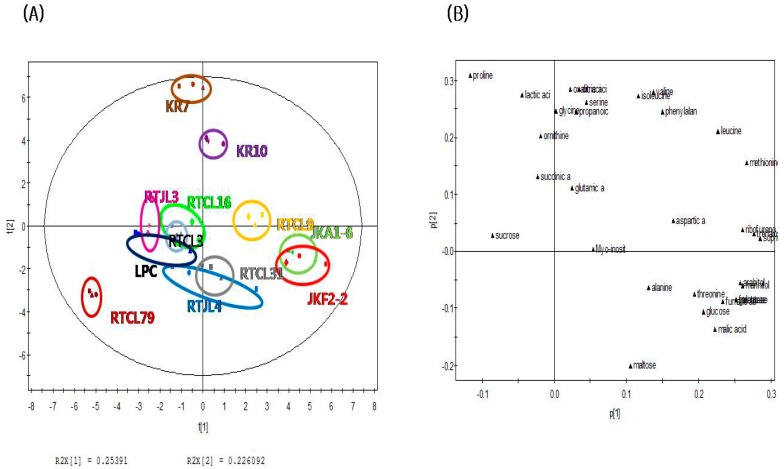
PCA plots of the nonvolatile volatile compounds identified in fermented rice according to strains of LABs: (**A**) score plot, (**B**) loading plot.

The PCA score plot of nonvolatile compounds in Figure 2A shows that rice samples fermented by obligatorily heterofermentative LAB such as JKA1-6, JKF2-2, and RTCL9 could be distinguished from the other samples (except for KR10, KR7, and RTJL4) fermented with obligatorily homofermentative/facultatively heterofermentative LABs along the PC1 axis (explaining 25.40% of the variance). Also, in contrast to the score plot for nonvolatile compounds, RTCL16 was not clearly separated from the other samples along the PC2 axis (explaining 22.6% of the variance). The PCA loading plot of nonvolatile compounds in Figure 2B shows that most carbohydrates (except for sucrose and maltose) were strongly associated with rice samples fermented by obligatorily heterofermentative LABs (KR10, KR7, RTCL9, JKA1-6, and JKF2-2) on the positive dimension of the PC1 axis of the PCA score plot. On the other hand, some amino acids (serine, valine, isoleucine, phenylalanine, glycine, and ornithine) and organic acids (lactic acid, succinic acid, propanoic acid, oxalic acid, and citric acid) were mainly responsible for rice samples fermented by heterofermentative LABs (KR10 and KR7) on the positive axis of the PCA score plot along the PC2 dimension. Thus, these compounds could be major nonvolatile compounds related to rice samples fermented by the obligatorily heterofermentative LABs (KR10, KR7, RTCL9, JKA1-6, and JKF2-2).

## 3. Materials and Methods

### 3.1. Chemicals and Reagents

Three internal standard compounds (2,3,5-trimethylpyrazine, 2,3-pentanedione, and tropic acid) and derivatization reagents [methoxyamine hydrochloride, pyridine, *N*,*O*-bis(trimethylsilyl)-tirfluoroacetamide (BSTFA) containing 1% trimethylchlorosilane (TMCS)] were purchased from Sigma-Aldrich (St. Louis, MO, USA). l-Threitol was obtained from Tokyo Chemical Industry (Tokyo, Japan), and l-4-hydroxyproline was obtained from Fluka Chemical (Milwaukee, WI, USA). Solvents such as methanol, chloroform, and water were of analytical grade (J.T. Baker, Phillipsburg, NJ, USA).

### 3.2. Sample Preparation

Milled rice was ground using a miller into 170 mesh. The distilled water was added at the ratio of 2.3:7.7 (ground rice: water, *w*/*w*) before the ground samples were treated with commercial 0.05% (*w*/*w*) α-amylase (BAN480L; Novozymes) and 0.05% (*w*/*w*) glucoamylase (AMZ1100; Novozymes, Bagsvaerd, Denmark). The enzyme saccharification was performed by shaking at 100 rpm and 63 °C for 20 h, following the previous method of Lee et al. [13]. Then, enzyme deactivation was performed at 85 °C for 30 min. After enzyme deactivation, samples were inoculated with 1% (*w*/*w*) of 12 different LABs (10^7^ CFU/mL), respectively (Table 3). For the present study, 12 LAB strains were isolated and selected from various Korean fermented foods, considering their tolerances to acid, salt, and glucose in a preliminary study (Table 3). Then, inoculated samples were fermented at 30 °C for 24 h. All samples were kept at about −70 °C in a deep freezer (I1 Shin Bio Base; Model No. DF8514; Dongdoocheon-si, Gyeonggido, Korea). All experiments were conducted in triplicate.

### 3.3. Extraction and Analysis of Volatile Compounds by Gas Chromatography-Mass Spectrometry

Solid phase micro-extraction (SPME) was used to obtain volatile profiles of fermented rice samples. Four g of fermented rice was put into a 20 mL screw vial with a screw cap (Ultraclean 18 mm, Agilent Technologies, Santa Clara, CA, USA). After sample preparation, the vial was maintained at 30 °C for 10 min to reach an equilibrium state. Volatiles in headspace were adsorbed onto SPME fiber coated with carboxen/polydimethylsiloxane/divinylbenzene (CAR/PDMS/DVB) (Supelco, Bellefonte, PA, USA). The adsorption and desorption conditions were the same as those used previously [2]. 2,3,5-Trimethylpyrazine and 2,3-pentanedione (100 mg/L in methanol) were used as internal standards of aromatic and aliphatic compounds, respectively.

Gas chromatography-mass spectrometry (GC-MS) analysis was performed using an HP 7890B GC system coupled to the 5977A mass selective detector (Agilent Technologies) and the multi-purpose sampler MPS 2 (Gerstel, Mülheim an der Ruhr, Germany) equipped with a DB-WAX capillary column (30 m length × 0.25 mm i.d. × 0.25 μm film thickness, J&W Scientific, Folsom, CA, USA). Other conditions for GC-MS analysis were based on a minor modification of Lee et al. [2].

### 3.4. Extraction and Analysis of Nonvolatile Compounds by Gas Chromatography-Time of Flight-Mass Spectrometry

One g of fermented rice was immersed in liquid nitrogen and then extracted with 20 mL of 80% methanol (J. T. Baker., Phillipsburg, NJ, USA) at 70 °C for 25 min in an ultrasonicator (Branson, Danbury, CT, USA). After that, it stayed in room temperature for 30 min, followed by an addition of 2 mL chloroform (J. T. Baker., Phillipsburg, NJ, USA). It was then sonicated for 20 min prior to a centrifugation for 10 min. Finally, 100 µL of the extracted layer was transferred to a 1.5 mL tube (Eppendorf, Hamburg, Germany) and then injected using internal standard followed by drying overnight in a centri-vap (Labconco Co., Kansas City, MO, USA). For methoximation, 50 µL of methoxyamine hydrochloride (20 mg/mL in pyridine) was added to the dried extract at 30 °C for 90 min. After that, derivatization for silylation was performed by 90 µL of *N*,*O*-bis (trimethylsilyl)-tirfluoroacetamide (BSTFA) containing 1% trimethylchlorosilane (TMCS), following the previous method of Son et al. [47]. Internal standard compounds were l-threitol (100 mg/L in water) for carbohydrates, l-4-hydroxyproline (100 mg/L in water) for amino acids, and tropic acid (100 mg/L in water) for organic acids, respectively.

Gas chromatography-time-of-flight/mass spectrometry (GC-TOF/MS) analysis was performed by the Agilent 6890N GC system (Agilent Technologies) coupled to the Leco Pegasus III mass spectrometer (Leco, St. Joseph, MI, USA) equipped with a DB-5MS column (30 m length × 0.25 mm i.d. × 0.25 μm film thickness, J&W Scientific). Other conditions for GC-TOF/MS analysis were based on a minor modification of Son et al. [47].

### 3.5. Identification and Semi-Quantification of Volatile and Nonvolatile Compounds

Volatile compounds were identified based on a comparison of the mass spectra in the NIST08 and Wiley 9 mass spectral libraries (Agilent Technologies, Palo Alto, CA, USA) and retention index (RI) values. The RI values of volatile compounds were calculated with an alkane mixture from C_7_ to C_30_ as external standards. In addition, volatile compounds were positively confirmed by comparing their mass spectrum and retention time with those of standard compounds. Nonvolatile compounds were identified by comparing their mass spectral data based on Fiehn library, replibrary, mainlibrary, Wiley 9, and in-house library, and then confirmed by comparing their mass spectral data and retention times to those of authentic standard compounds. The identification and semi-quantification procedures were the same as those used previously [47].

### 3.6. Statistical Analysis

The ANOVA was performed with general linear model procedure using SPSS (version 12.0, Chicago, IL, USA). Duncan’s multiple comparison test (*p* < 0.05) was applied to identify statistically significant differences. PCA was performed to discriminate fermented rice samples on the basis of their volatile and nonvolatile compounds profiles according to microbial strains using SIMCA-P (version 11.0, Umetrics, Umea, Sweden). 

## 4. Conclusions

This study investigated differences in the volatile and nonvolatile compounds of rice samples fermented by 12 different LABs. The level of ethanol differed significantly between obligatorily heterofermentative and facultatively heterofermentative/obligatorily homofermentative LAB samples. In homofermentative LAB, ethanol comes from pyruvate via acetyl-CoA, whereas it is derived from the pentose phosphate pathway of glucose in heterofermentative LAB. In addition, the amounts of 3-hydroxybutan-2-one and butane-2,3-dione were larger for the homofermentative/facultatively heterofermentative LABs RTJL3, LPC, RTCL16, RTCL79, and RTCL31 than for the obligatorily heterofermentative LABs KR10, KR7, RTJL4, JFK2-2, JKA1-6, and RTCL9. This difference could at least partially be due to low-ethanol-tolerant microorganisms preferentially switching pyruvate utilization to the acetoin biosynthetic pathway in order to dispose of ethanol present at toxic levels.

The application of PCA to data sets of the profiles of volatile and nonvolatile compounds revealed that fermented rice samples can be distinguished according to different LAB strains. The volatile compounds 2-ethylfuran, 2-propylfuran, 2-butylfuran, 2-pentylfuran, hexanal, 2-methylbutanal, pentan-1-ol, and butan-1-ol were strongly correlated with the rice sample fermented by *Lac. sakei* (RTCL16). On the other hand, ketones and aldehydes such as butane-2,3-dione, 3-hydroxybutan-2-one, 6-methylhept-5-en-2-one, 2-methylbutanal, and nonanal were related to fermented rice samples inoculated with obligatorily heterofermentative LAB. These findings indicate that the profiles of volatile and nonvolatile compounds of fermented rice inoculated with different LABs can change significantly depending on the microbial strains present during the fermentation process. These results can be used to improve the quality of rice-based fermented products and develop rice-based functional foods, such as probiotic beverages.

## Figures and Tables

**Table 1:** Volatile compounds identified in fermented rice according to strains of lactic acid bacteria (LABs).

No.	Compounds	RI ^1^	Relative Peak Area ^2^												ID ^3^
			RTJL3	LPC	KR10	KR7	RTCL16	RTCL79	RTJL4	RTCL3	RTCL31	JFK2-2	JKA1-6	RTCL	
**ACIDS**															
1	acetic acid	1457	4.39 ± 0.69 ab ^4^	1.45 ± 0.42 a	9.99 ± 1.85 ab	13.42 ± 8.76 ab	80.20 ± 19.99 c	11.25 ± 8.77 ab	8.48 ± 4.02 ab	14.36 ± 4.06 ab	1.47 ± 0.71 a	18.36 ± 8.57 b	14.87 ± 2.23 ab	18.39 ± 6.29 b	A
2	hexanoic acid	1860	0.95 ± 0.06 g	0.45 ± 0.08 cd	0.61 ± 0.08 de	N.D. a	2.17 ± 0.29 h	0.36 ± 0.14 bc	0.48 ± 0.10 cd	0.89 ± 0.12 f	0.24 ± 0.08 b	0.78 ± 0.10 efg	0.59 ± 0.04 de	0.70 ± 0.06 ef	A
3	octanoic acid	2065	0.14 ± 0.01 b	0.34 ± 0.02 d	0.04 ± 0.01 a	0.14 ± 0.03 b	0.17 ± 0.02 bc	0.16 ± 0.05 bc	0.22 ± 0.04 c	0.49 ± 0.12 e	0.14 ± 0.01 b	0.05 ± 0.01 a	0.03 ± 0.01 a	0.43 ± 0.04 e	A
**ALDEHYDES**															
4	2-methylbutanal	911	N.D. ^5^ a	N.D. a	N.D. a	N.D. a	0.64 ± 0.08 d	N.D. a	N.D. a	0.34 ± 0.02 c	0.07 ± 0.02 b	N.D. a	N.D. a	N.D. a	A
5	3-methylbutanal	915	0.42 ± 0.04 c	0.34 ± 0.05 b	N.D. a	N.D. a	N.D. a	N.D. a	N.D. a	0.58 ± 0.03 d	N.D. a	N.D. a	N.D. a	N.D. a	A
6	hexanal	1078	N.D. a	N.D. a	0.52 ± 0.34 b	0.64 ± 0.21 b	4.08 ± 0.11 d	N.D. a	N.D. a	3.02 ± 0.10 c	0.72 ± 0.04 b	0.51 ± 0.12 b	0.22 ± 0.01 a	0.19 ± 0.01 a	A
7	nonanal	1397	N.D. a	N.D. a	N.D. a	N.D. a	N.D. a	0.168 ± 0.018 b	N.D. a	N.D. a	0.218 ± 0.060 c	N.D. a	N.D. a	N.D. a	A
8	benzaldehyde	1530	2.68 ± 0.22 c	2.72 ± 0.02 c	0.40 ± 0.05 a	0.52 ± 0.06 a	6.94 ± 0.54 e	0.47 ± 0.05 a	0.24 ± 0.05 a	3.19 ± 0.04 d	2.13 ± 0.12 b	0.28 ± 0.05 a	0.33 ± 0.03 a	0.38 ± 0.03 a	A
**ESTERS**															
9	ethyl acetate	885	0.33 ± 0.03 a	0.25 ± 0.06 a	0.66 ± 0.02 bc	0.97 ± 0.13 d	0.72 ± 0.09 c	0.34 ± 0.12 a	0.96 ± 0.02 d	0.37 ± 0.12 a	0.25 ± 0.03 a	0.70 ± 0.04 bc	1.71 ± 0.07 e	0.56 ± 0.05 b	A
10	methyl butanoate	984	0.13 ± 0.02 a	0.13 ± 0.01 a	0.21 ± 0.02 bc	0.27 ± 0.02 d	0.43 ± 0.06 e	0.19 ± 0.02 bc	0.19 ± 0.03 bc	0.23 ± 0.02 cd	0.18 ± 0.01 ab	0.18 ± 0.04 ab	0.22 ± 0.02 bcd	0.21 ± 0.01 bc	B
11	butyl acetate	1070	N.D. a	N.D. a	0.09 ± 0.05 b	0.13 ± 0.01 c	N.D. a	N.D. a	N.D. a	N.D. a	N.D. a	0.13 ± 0.01 c	0.18 ± 0.02 d	0.16 ± 0.03 cd	B
12	hexyl acetate	1274	N.D. a	N.D. a	0.07 ± 0.01 b	0.12 ± 0.01 c	N.D. a	N.D. a	N.D. a	N.D. a	N.D. a	N.D. a	N.D. a	N.D. a	A
**KETONES**															
13	propan-2-one	816	2.14 ± 0.43 cd	1.93 ± 0.15 c	2.69 ± 0.29 def	N.D. a	6.90 ± 0.17 g	2.45 ± 0.26 de	N.D. a	2.84 ± 0.18 ef	1.93 ± 0.14 c	1.00 ± 0.20 b	1.36 ± 0.06 b	1.13 ± 0.11 b	A
14	butan-2-one	901	0.52 ± 0.08 d	0.55 ± 0.03 d	N.D. a	N.D. a	1.57 ± 0.05 f	0.59 ± 0.03 d	N.D. a	0.69 ± 0.03 e	0.56 ± 0.02 d	0.39 ± 0.02 c	0.38 ± 0.08 c	0.29 ± 0.07 b	A
15	butane-2,3-dione	975	67.10 ± 3.89 e	29.33 ± 1.30 d	0.08 ± 0.01 a	0.19 ± 0.04 a	7.04 ± 0.64 c	2.66 ± 0.22 ab	N.D. a	3.77 ± 0.21 b	1.98 ± 0.02 ab	0.44 ± 0.03 a	0.14 ± 0.02 a	0.09 ± 0.01 a	A
16	3-hydroxybutan-2-one	1284	8.80 ± 0.35 c	3.12 ± 0.33 b	N.D. a	N.D. a	8.64 ± 0.96 c	2.88 ± 0.42 b	N.D. a	2.70 ± 0.35 b	3.15 ± 0.42 b	N.D. a	N.D. a	N.D. a	A
17	6-methylhept-5-en-2-one	1340	0.15 ± 0.03 c	0.29 ± 0.02 d	N.D.	N.D. a	N.D. a	0.13 ± 0.01 b	N.D. a	0.31 ± 0.02 e	0.11 ± 0.01 b	N.D. a	N.D. a	N.D. a	A
18	nonan-2-one	1390	0.22 ± 0.01 d	0.14 ± 0.03 c	0.34 ± 0.03 e	0.42 ± 0.05 f	0.37 ± 0.05 e	0.16 ± 0.01 a	N.D. a	0.15 ± 0.01 c	0.06 ± 0.01 b	N.D. a	N.D. a	N.D. a	A
**ALCOHOLS**															
19	ethanol	938	7.28 ± 0.87 a	7.46 ± 0.42 a	148.13 ± 6.73 d	171.54 ± 24.01 e	20.79 ± 1.95 a	7.29 ± 0.40 a	88.28 ± 14.58 bc	9.52 ± 1.64 a	7.39 ± 0.46 a	101.64 ± 7.36 c	82.42 ± 1.90 b	85.02 ± 8.11 b	A
20	butan-1-ol	1151	0.24 ± 0.02 a	0.18 ± 0.02 a	0.36 ± 0.02 c	0.19 ± 0.01 a	0.90 ± 0.09 d	0.35 ± 0.03 bc	0.19 ± 0.03 a	0.38 ± 0.03 c	0.21 ± 0.01 a	0.34 ± 0.02 bc	0.38 ± 0.01 c	0.30 ± 0.01 b	A
21	3-methylbutan-1-ol	1212	0.60 ± 0.04 ab	0.49 ± 0.03 a	1.11 ± 0.03 f	1.36 ± 0.21 g	1.78 ± 0.12	0.73 ± 0.08 bc	0.76 ± 0.05 cd	0.68 ± 0.04 bc	0.57 ± 0.02 ab	0.99 ± 0.01 ef	0.90 ± 0.01 de	0.93 ± 0.15 e	A
22	pentan-1-ol	1255	1.32 ± 0.28 ab	1.47 ± 0.15 abc	1.42 ± 0.05 ab	1.73 ± 0.11 c	4.04 ± 0.13 d	1.18 ± 0.20 a	1.36 ± 0.06 ab	1.56 ± 0.14 bc	1.55 ± 0.20 bc	1.36 ± 0.21 ab	1.29 ± 0.04 ab	1.23 ± 0.08 a	A
23	heptan-2-ol	1325	N.D. a	N.D. a	0.44 ± 0.02 d	0.68 ± 0.10 e	N.D. a	N.D. a	0.31 ± 0.04 b	N.D. a	N.D. a	0.39 ± 0.08 cd	0.41 ± 0.04 d	0.33 ± 0.06 bc	A
24	hexan-1-ol	1358	4.18 ± 0.26 a	4.25 ± 0.19 a	8.04 ± 0.45 c	10.17 ± 0.19 d	20.11 ± 1.36 e	6.91 ± 0.24 b	7.51 ± 0.38 bc	7.55 ± 0.49 bc	7.58 ± 0.45 bc	7.22 ± 0.43 bc	6.92 ± 0.06 b	7.20 ± 0.05 bc	A
25	oct-1-en-3-ol	1454	0.76 ± 0.10 b	0.89 ± 0.03 b	0.91 ± 0.26 b	1.27 ± 0.35 c	N.D. a	0.66 ± 0.22 b	0.77 ± 0.16 b	0.82 ± 0.28 b	0.84 ± 0.03 b	N.D. a	N.D. a	N.D. a	A
26	2-ethylhexan-1-ol	1495	0.09 ± 0.01 a	0.08 ± 0.01 a	0.20 ± 0.01 cd	0.44 ± 0.03 e	0.39 ± 0.07 e	0.09 ± 0.01 a	0.12 ± 0.02 ab	0.24 ± 0.05 d	0.08 ± 0.01 a	0.16 ± 0.04 bc	0.26 ± 0.01 d	0.21 ± 0.03 cd	A
27	(*E*)-hept-2-en-1-ol	1517	N.D. a	N.D. a	0.11 ± 0.01 b	0.15 ± 0.03 c	N.D. a	N.D. a	0.15 ± 0.01 c	N.D. a	N.D. a	0.13 ± 0.01 b	0.12 ± 0.01 b	0.1 ± 0.02 b	A
28	octan-1-ol	1564	0.26 ± 0.03 ab	0.24 ± 0.02 a	0.37 ± 0.02 c	0.45 ± 0.10 d	0.75 ± 0.05 e	0.28 ± 0.04 ab	0.25 ± 0.02 ab	0.33 ± 0.02 bc	0.25 ± 0.04 ab	0.32 ± 0.01 bc	0.32 ± 0.04 bc	0.29 ± 0.03 ab	A
29	(*E*)-oct-2-en-1-ol	1616	N.D. a	N.D. a	0.05 ± 0.01 b	0.06 ± 0.01 c	N.D. a	N.D. a	N.D. a	N.D. a	N.D. a	N.D. a	N.D. a	N.D. a	A
30	nonan-1-ol	1667	0.33 ± 0.04 ab	0.30 ± 0.02 a	0.40 ± 0.05 bc	0.59 ± 0.09 d	1.03 ± 0.07 e	0.36 ± 0.04 abc	0.34 ± 0.04 ab	0.43 ± 0.03 c	0.31 ± 0.04 a	0.40 ± 0.01 bc	0.41 ± 0.02 bc	0.3 ± 0.03 abc	A
31	butane-1,4-diol	1928	0.19 ± 0.01 ab	0.22 ± 0.03 ab	0.49 ± 0.07 e	0.77 ± 0.10 f	0.54 ± 0.04 e	0.19 ± 0.03 ab	0.14 ± 0.01 a	0.31 ± 0.06 cd	0.18 ± 0.01 a	0.31 ± 0.05 cd	0.3 ± 0.04 d	0.27 ± 0.02 bc	A
**FURAN DERIVATIVES**															
32	2-ethylfuran	950	1.23 ± 0.34 d	1.16 ± 0.13 cd	0.57 ± 0.09 ab	0.90 ± 0.10 bc	2.40 ± 0.08 f	1.16 ± 0.11 cd	0.84 ± 0.08 ab	1.54 ± 0.38 e	0.58 ± 0.17 ab	0.55 ± 0.11 a	0.56 ± 0.11 a	0.58 ± 0.07 ab	A
33	2-propylfuran	1030	1.59 ± 0.22 e	1.45 ± 0.11 cde	1.32 ± 0.08 bcd	1.88 ± 0.10 f	3.15 ± 0.19 g	1.51 ± 0.17 de	1.32 ± 0.08 bcd	2.02 ± 0.14 f	0.86 ± 0.19 a	1.25 ± 0.09 bc	1.33 ± 0.08 bcd	1.10 ± 0.05 b	A
34	2-butylfuran	1130	3.48 ± 0.30 b	3.06 ± 0.09 ab	3.07 ± 0.06 ab	4.20 ± 0.47 c	8.93 ± 0.17 d	3.40 ± 0.43 b	3.24 ± 0.08 ab	4.41 ± 0.16 c	3.27 ± 0.72 ab	2.94 ± 0.02 ab	3.22 ± 0.19 ab	2.76 ± 0.05 a	A
35	2-pentylfuran	1231	16.15 ± 1.55 bc	13.51 ± 0.78 ab	12.82 ± 0.46 a	16.97 ± 1.75 c	49.08 ± 0.93 e	16.05 ± 2.59 bc	14.77 ± 1.06 abc	20.05 ± 1.59 d	13.32 ± 2.52 a	12.93 ± 0.37 a	14.32 ± 0.82 abc	12.69 ± 0.59 a	B
**BENZENE AND BENZENE DERIVATIVES**															
36	toluene	1036	1.32 ± 0.12 abc	1.01 ± 0.01 a	2.06 ± 0.25 de	2.39 ± 0.10 e	2.33 ± 0.26 e	1.11 ± 0.15 a	2.33 ± 0.18 e	1.73 ± 0.50 bcd	1.28 ± 0.16 ab	1.79 ± 0.18 cd	1.8 ± 0.09 d	2.00 ± 0.60 de	A
37	ethylbenzene	1120	2.23 ± 0.37 ab	1.93 ± 0.12 ab	2.10 ± 0.13 ab	2.47 ± 0.40 ab	4.51 ± 0.07 d	2.03 ± 0.19 ab	2.34 ± 0.32 ab	2.52 ± 0.05 b	3.88 ± 0.78 c	1.93 ± 0.12 ab	1.96 ± 0.15 ab	1.90 ± 0.15 a	B
38	1,4-xylene	1136	1.63 ± 0.15 ab	1.45 ± 0.05 a	1.82 ± 0.13 abc	2.17 ± 0.41 cd	3.55 ± 0.20 e	1.52 ± 0.07 a	1.69 ± 0.09 ab	1.97 ± 0.11 bc	2.49 ± 0.45 d	1.68 ± 0.15 ab	1.67 ± 0.08 ab	1.63 ± 0.08 ab	A
39	1,2-xylene	1179	N.D. a	N.D. a	0.80 ± 0.05 b	1.14 ± 0.11 c	N.D. a	N.D. a	N.D. a	N.D. a	N.D. a	0.61 ± 0.49 b	0.83 ± 0.04 b	0.89 ± 0.18 bc	A
40	propylbenzene	1205	N.D. a	0.04 ± 0.01 ab	0.05 ± 0.01 bc	0.04 ± 0.00 ab	0.15 ± 0.01 e	0.06 ± 0.01	0.10 ± 0.02 d	0.07 ± 0.01 bcd	0.09 ± 0.01 cd	N.D. bcd	0.09 ± 0.06 cd	N.D. a	B
41	1-ethyl-2-methylbenzene	1222	0.04 ± 0.01 a	0.0 ± 0.01 a	0.14 ± 0.02 d	0.09 ± 0.02 c	0.30 ± 0.01 e	0.04 ± 0.01 a	0.06 ± 0.01 ab	0.05 ± 0.01 ab	0.09 ± 0.01 c	0.07 ± 0.01 bc	0.06 ± 0.01 ab	0.16 ± 0.01 d	B
42	1-ethyl-3-methylbenzene	1223	0.13 ± 0.00 bc	0.16 ± 0.05 c	0.07 ± 0.00 a	0.17 ± 0.01 c	0.13 ± 0.02 bc	0.10 ± 0.01 ab	0.16 ± 0.04 c	0.14 ± 0.02 bc	0.23 ± 0.06 d	0.14 ± 0.01 bc	0.14 ± 0.01 bc	0.07 ± 0.01 a	A
43	1,3,5-trimethylbenzene	1242	0.13 ± 0.00 c	0.12 ± 0.02 c	0.19 ± 0.02 d	N.D. a	0.13 ± 0.01 c	N.D. a	0.13 ± 0.02 c	0.13 ± 0.01 c	0.20 ± 0.04 d	0.15 ± 0.03 c	0.07 ± 0.02 b	0.08 ± 0.02 b	B
44	1,2,4-trimethylbenzene	1280	N.D. a	0.53 ± 0.01 ab	N.D. a	1.18 ± 1.02 b	N.D. a	0.56 ± 0.05 ab	N.D. a	N.D. a	N.D. a	N.D. b	N.D. a	N.D. a	A
45	1,2,3-trimethylbenzene	1337	0.04 ± 0.00 ab	0.03 ± 0.00 ab	N.D. a	N.D. a	N.D. a	0.04 ± 0.02 ab	0.18 ± 0.03 c	0.05 ± 0.01 ab	0.07 ± 0.03 b	0.19 ± 0.05 c	0.19 ± 0.07 c	N.D. a	A
**HYDROCARBONS**															
46	octane	780	0.98 ± 0.17 ab	0.76 ± 0.04 a	1.19 ± 0.16 bc	1.41 ± 0.08 cd	3.16 ± 0.25 e	1.06 ± 0.16 ab	1.62 ± 0.43 d	1.10 ± 0.19 ab	0.83 ± 0.16 ab	1.00 ± 0.18 ab	0.87 ± 0.08 ab	0.74 ± 0.11 a	A
**TERPENES**															
47	1-methyl-4-prop-1-en-2-ylcyclohexene (limonene)	1194	N.D. a	N.D. a	0.33 ± 0.06 c	N.D. a	N.D. a	N.D. a	N.D. a	N.D. a	N.D. a	N.D. a	N.D. a	0.15 ± 0.03 b	A

^1^ Retention indices were determined using n-alkanes C_7_ to C_30_; ^2^ Mean values of relative peak area to that of internal standard ± standard deviation; ^3^ Identification: A, mass spectrum agreed with the authentic compound; B, mass spectrum and retention index were consistent with those of Wiley library and literatures; ^4^ There were significant differences (*p* < 0.05) among 12 different samples using Duncan’s multiple comparison test between the samples with the different letter in a row; ^5^ Not detected.

**Table 2:** Nonvolatile compounds identified in fermented rice according to strains of LABs.

No.	Compounds	Relative Peak Area ^1^												ID ^2^
		RTJL3	LPC	KR10	KR7	RTCL16	RTCL79	RTJL4	RTCL3	RTCL31	JFK2-2	JKA1-6	RTCL	
**AMINO ACIDS**														
1	alanine	0.43 ± 0.05 f ^3^	0.36 ± 0.02 e	0.16 ± 0.02 b	0.22 ± 0.02 c	0.55 ± 0.01 cg	0.08 ± 0.02 a	0.24 ± 0.03 c	0.05 ± 0.01 a	0.44 ± 0.03 f	0.38 ± 0.02 e	0.47 ± 0.02 f	0.31 ± 0.04 d	A
2	asparagine	N.D. ^4^ a	N.D. a	0.03 ± 0.00 d	0.03 ± 0.01 cd	N.D. a	0.01 ± 0.00 b	0.02 ± 0.00 c	0.02 ± 0.00 c	0.02 ± 0.00 c	0.01 ± 0.00 b	0.02 ± 0.01 c	0.02 ± 0.00 b	A
3	citrulline	0.03 ± 0.00 b	0.03 ± 0.00 b	N.D. a	N.D. a	0.04 ± 0.01 c	0.04 ± 0.00 c	N.D. a	N.D. a	0.07 ± 0.01 e	0.05 ± 0.01 d	0.05 ± 0.01 d	N.D. a	A
4	γ-aminobutyric acid	0.05 ± 0.01 a	0.09 ± 0.00 d	0.09 ± 0.00 d	0.64 ± 0.02 g	0.09 ± 0.00 de	0.07 ± 0.01 b	0.16 ± 0.01 f	0.08 ± 0.00 cd	0.08 ± 0.01 cd	0.09 ± 0.00 d	0.08 ± 0.01 cd	0.08 ± 0.00 cd	A
5	tryptophan	0.01 ± 0.00 b	N.D. a	0.03 ± 0.01 c	0.02 ± 0.00 c	0.01 ± 0.00 b	N.D. a	N.D. a	0.02 ± 0.00 c	N.D. a	0.02 ± 0.01 c	0.02 ± 0.00 c	0.02 ± 0.00 c	A
6	valine	0.21 ± 0.02 e	0.11 ± 0.00 b	0.29 ± 0.01 b	0.25 ± 0.01 g	0.23 ± 0.00 f	0.01 ± 0.00 a	0.11 ± 0.00 b	0.19 ± 0.00 d	0.17 ± 0.00 c	0.16 ± 0.00 c	0.18 ± 0.01 d	0.23 ± 0.01 f	A
7	leucine	0.20 ± 0.02 c	0.14 ± 0.01 b	0.50 ± 0.01 j	0.40 ± 0.01 g	0.43 ± 0.01 i	0.01 ± 0.01 a	0.20 ± 0.00 c	0.35 ± 0.01 e	0.27 ± 0.00 d	0.37 ± 0.01 f	0.40 ± 0.00 gh	0.42 ± 0.03 hi	A
8	isoleucine	0.03 ± 0.01 c	N.D. a	0.12 ± 0.00 g	0.08 ± 0.00 ef	0.08 ± 0.00 e	N.D. a	N.D. a	0.02 ± 0.01 b	0.02 ± 0.00 b	0.03 ± 0.00 c	0.05 ± 0.00 d	0.09 ± 0.00 f	A
9	proline	0.36 ± 0.03 e	0.31 ± 0.01 d	0.46 ± 0.01 g	0.44 ± 0.01 f	0.27 ± 0.01 c	0.27 ± 0.01 c	0.23 ± 0.01 b	0.27 ± 0.01 c	0.25 ± 0.01 c	0.22 ± 0.01 ab	0.21 ± 0.01 a	0.22 ± 0.002 ab	A
10	glycine	0.13 ± 0.05 b	0.23 ± 0.02 c	0.20 ± 0.01 c	0.19 ± 0.02 c	0.10 ± 0.08 b	0.04 ± 0.01 a	0.04 ± 0.01 a	0.11 ± 0.01 b	0.09 ± 0.00 b	0.11 ± 0.00 b	0.11 ± 0.01 b	0.10 ± 0.01 b	A
11	serine	0.24 ± 0.07 efg	0.20 ± 0.02 cde	0.26 ± 0.01 g	0.26 ± 0.02 fg	0.15 ± 0.01 ab	0.15 ± 0.01 ab	0.16 ± 0.02 abc	0.12 ± 0.01 a	0.17 ± 0.02 bc	0.18 ± 0.01 bcd	0.20 ± 0.02 cde	0.22 ± 0.012 def	A
12	threonine	0.04 ± 0.01 def	0.01 ± 0.00 a	0.03 ± 0.00 cd	0.02 ± 0.00 ab	0.04 ± 0.00 gh	0.02 ± 0.01 bc	0.02 ± 0.01 ab	0.04 ± 0.01 efg	0.03 ± 0.00 de	0.05 ± 0.00 h	0.04 ± 0.00 fgh	0.05 ± 0.00 h	A
13	methionine	0.03 ± 0.00 bc	0.03 ± 0.01 bc	0.04 ± 0.00 e	0.03 ± 0.00 cd	0.03 ± 0.00 bc	N.D. a	0.03 ± 0.00 bc	0.03 ± 0.00 b	0.02 ± 0.00 b	0.04 ± 0.01 e	0.04 ± 0.01 de	0.04 ± 0.00 cd	A
14	aspartic acid	0.16 ± 0.02 e	0.12 ± 0.02 d	0.09 ± 0.00 c	0.06 ± 0.01 b	0.08 ± 0.004 c	N.D. a	N.D. a	0.10 ± 0.00 c	0.06 ± 0.00 b	0.15 ± 0.01 e	0.13 ± 0.00 d	0.15 ± 0.01 e	A
15	glutamic acid	0.08 ± 0.00 c	0.08 ± 0.00 c	0.13 ± 0.00 d	0.02 ± 0.00 ab	0.07 ± 0.01 c	N.D. a	N.D. a	0.07 ± 0.01 c	0.06 ± 0.00 c	0.03 ± 0.05 b	0.05 ± 0.00 c	0.08 ± 0.01 c	A
16	phenylalanine	0.05 ± 0.00 b	0.04 ± 0.00 b	0.11 ± 0.00 e	0.10 ± 0.01 de	0.08 ± 0.01 cd	N.D. a	0.05 ± 0.01 b	0.08 ± 0.01 cd	N.D. a	0.06 ± 0.05 bc	0.08 ± 0.00 cde	0.08 ± 0.00 cde	A
17	ornithine	N.D. a	N.D. a	0.44 ± 0.03 d	0.44 ± 0.01 a	0.01 ± 0.01 a	N.D. d	0.33 ± 0.02 d	0.35 ± 0.00 a	0.27 ± 0.02 a	N.D. c	N.D. c	N.D. b	A
**ORGANIC ACIDS**														
18	lactic acid	122.46 ± 3.81 cd	151.00 ± 10.23 d	107.17 ± 6.14 cd	991.52 ± 92.63 e	39.93 ± 0.69 a	146.83 ± 9.76 d	71.80 ± 4.81 abc	110.65 ± 8.23 cd	53.92 ± 2.68 ab	92.52 ± 8.17 bc	70.19 ± 3.22 abc	72.45 ± 5.00 abc	A
19	citric acid	0.09 ± 0.01 b	N.D. a	0.24 ± 0.00 cd	2.05 ± 0.06 f	N.D. a	N.D. a	0.21 ± 0.02 c	0.22 ± 0.01 c	N.D. a	0.27 ± 0.04 de	0.30 ± 0.02 e	0.26 ± 0.01 de	A
20	propanoic acid	0.06 ± 0.01 cd	0.05 ± 0.01 cd	0.03 ± 0.00 ab	0.27 ± 0.03 f	0.04 ± 0.01 abc	0.03 ± 0.01 ab	0.04 ± 0.01 bcd	0.02 ± 0.00 a	0.02 ± 0.01 a	0.09 ± 0.02 e	0.06 ± 0.01 d	0.03 ± 0.01 ab	A
18	lactic acid	122.46 ± 3.81 cd	151.00 ± 10.23 d	107.17 ± 6.14 cd	991.52 ± 92.63 e	39.93 ± 0.69 a	146.83 ± 9.76 d	71.80 ± 4.81 abc	110.65 ± 8.23 cd	53.92 ± 2.68 ab	92.52 ± 8.17 bc	70.19 ± 3.22 abc	72.45 ± 5.00 abc	A
19	citric acid	0.09 ± 0.01 b	N.D. a	0.24 ± 0.00 cd	2.05 ± 0.06 f	N.D. a	N.D. a	0.21 ± 0.02 c	0.22 ± 0.01 c	N.D. a	0.27 ± 0.04 de	0.30 ± 0.02 e	0.26 ± 0.01 de	A
20	propanoic acid	0.06 ± 0.01 cd	0.05 ± 0.01 cd	0.03 ± 0.00 ab	0.27 ± 0.03 f	0.04 ± 0.01 abc	0.03 ± 0.01 ab	0.04 ± 0.01 bcd	0.02 ± 0.00 a	0.02 ± 0.01 a	0.09 ± 0.02 e	0.06 ± 0.01 d	0.03 ± 0.01 ab	A
21	oxalic acid	0.05 ± 0.00 bc	0.05 ± 0.00 b	0.05 ± 0.01 b	0.43 ± 0.03 d	0.05 ± 0.01 b	N.D. a	0.06 ± 0.01 bc	0.05 ± 0.00 b	0.05 ± 0.00 bc	0.06 ± 0.01 bc	0.07 ± 0.00 c	0.06 ± 0.01 bc	A
22	succinic acid	0.07 ± 0.01 ab	0.22 ± 0.12 c	0.03 ± 0.00 a	0.24 ± 0.02 c	0.06 ± 0.06 ab	0.07 ± 0.01 ab	0.10 ± 0.01 b	0.05 ± 0.01 ab	0.03 ± 0.01 ab	0.05 ± 0.01 ab	0.03 ± 0.00 ab	0.30 ± 0.01 d	A
23	fumaric acid	N.D. a	N.D. a	N.D. a	N.D. a	0.03 ± 0.00 b	N.D. a	N.D. a	N.D. a	N.D. a	0.06 ± 0.00 c	0.06 ± 0.01 d	N.D. a	A
24	malic acid	N.D. a	N.D. a	N.D. a	N.D. a	N.D. a	N.D. a	0.28 ± 0.02 b	N.D. a	N.D. a	0.27 ± 0.03 b	0.32 ± 0.02 c	N.D. a	A
**CARBOHYDRATES**														
25	ribofuranose	N.D. a	N.D. a	1.88 ± 0.33 cd	1.32 ± 0.13 b	1.70 ± 0.40 bcd	N.D. a	1.84 ± 0.38 cd	1.35 ± 0.10 b	1.89 ± 0.33 cd	2.03 ± 0.29 e	1.60 ± 0.09 bc	1.63 ± 0.08 bcd	B
26	arabitol	0.02 ± 0.00 a	0.02 ± 0.00 a	0.05 ± 0.00 bc	0.05 ± 0.00 bcd	0.03 ± 0.00 a	0.04 ± 0.01 b	0.06 ± 0.01 e	0.05 ± 0.00 bcd	0.06 ± 0.00 de	0.07 ± 0.00 f	0.07 ± 0.01 f	0.05 ± 0.01 cde	A
27	xylose	0.07 ± 0.00	0.10 ± 0.04	0.14 ± 0.03	0.09 ± 0.01	0.13 ± 0.04	0.01 ± 0.00	0.13 ± 0.02	0.09 ± 0.01	0.14 ± 0.04	0.21 ± 0.07	0.12 ± 0.01	0.13 ± 0.00	A
28	*myo*-inositol	0.37 ± 0.09 b	0.41 ± 0.01 bcd	0.48 ± 0.01 d	0.44 ± 0.04 cd	0.45 ± 0.03 cd	0.43 ± 0.01 bcd	0.44 ± 0.06 cd	0.40 ± 0.01 bc	0.46 ± 0.017 cd	0.46 ± 0.03 cd	0.44 ± 0.00 cd	0.31 ± 0.00 a	A
29	sucrose	0.41 ± 0.03 bc	0.32 ± 0.03 a	0.55 ± 0.01 ef	0.54 ± 0.05 ef	0.45 ± 0.01 cd	0.51 ± 0.03 de	0.59 ± 0.08 f	0.59 ± 0.02 f	0.69 ± 0.04 g	0.31 ± 0.03 a	0.38 ± 0.02 ab	0.37 ± 0.07 ab	A
30	maltose	20.47 ± 1.12 b	15.34 ± 1.44 a	12.24 ± 0.82 a	21.79 ± 1.49 bc	22.45 ± 3.81 bc	24.67 ± 0.81 cd	29.39 ± 3.90 ef	24.92 ± 0.96 cd	32.78 ± 1.05 f	24.93 ± 1.60 cd	27.01 ± 3.96 de	25.28 ± 0.83 cd	A
31	trehalose	4.07 ± 0.47 b	2.67 ± 0.48 a	7.69 ± 0.25 e	7.75 ± 0.67 e	4.99 ± 0.18 c	5.70 ± 0.30 c	6.75 ± 0.89 d	6.74 ± 0.19 d	7.67 ± 0.22 e	9.86 ± 0.47 f	9.54 ± 0.09 f	10.01 ± 0.37 f	B
32	sophorose	4.99 ± 0.28 b	3.94 ± 0.658 a	7.34 ± 0.161 e	7.15 ± 0.56 de	5.60 ± 0.26 bc	5.81 ± 0.193 c	6.64 ± 0.96 de	6.40 ± 0.18 cd	7.32 ± 0.298 e	8.94 ± 0.55 f	8.56 ± 0.22 f	9.10 ± 0.39 f	B
33	mannose	82.23 ± 0.87 ab	93.91 ± 11.22 cd	85.02 ± 2.92 abcd	83.09 ± 3.32 abc	79.09 ± 5.55 a	76.00 ± 1.81 a	91.48 ± 12.40 bcd	83.10 ± 1.78 abc	93.37 ± 3.57 cd	94.44 ± 4.12 d	94.39 ± 4.28 d	93.65 ± 1.41 cd	A
34	fructose	23.30 ± 0.25 ab	26.61 ± 3.18 cd	24.09 ± 0.83 abcd	23.54 ± 0.94 abc	22.41 ± 1.57 a	21.53 ± 0.51 a	25.92 ± 3.51 bcd	23.54 ± 0.50 abc	26.46 ± 1.01 cd	26.76 ± 1.17 d	26.74 ± 1.21 d	26.53 ± 0.40 cd	A
35	galactose	17.82 ± 0.19 ab	20.35 ± 2.43 cd	18.42 ± 0.63 abcd	18.00 ± 0.72 abc	17.14 ± 1.20 a	16.47 ± 0.39 a	19.82 ± 2.69 bcd	18.00 ± 0.39 abc	20.23 ± 0.77 cd	20.46 ± 0.89 d	20.45 ± 0.93 d	20.30 ± 0.30 cd	A
36	glucose	60.56 ± 2.79 abc	70.09 ± 5.14 d	59.22 ± 2.69 ab	59.22 ± 2.05 ab	56.34 ± 3.70 a	54.54 ± 1.02 a	66.37 ± 8.78 bcd	59.43 ± 1.37 ab	67.10 ± 3.12 cd	66.68 ± 2.75 bcd	67.26 ± 4.68 cd	66.54 ± 3.10 bcd	A
37	mannitol	N.D. a	N.D. a	0.39 ± 0.04 b	0.45 ± 0.26 b	N.D. a	N.D. a	0.76 ± 0.11 c	N.D. a	N.D. a	3.13 ± 0.16 e	2.09 ± 0.04 d	N.D. a	A

^1^ Mean values of relative peak area to that of internal standard ± standard deviation; ^2^ Identification: A, mass spectrum agreed with the authentic compound; B, mass spectrum agreed with library; ^3^ There were significant differences (*p* < 0.05) among 12 different samples using Duncan’s multiple comparison test between the samples with the different letter in a row; ^4^ Not detected.

**Table 3:** The abbreviation of fermented rice samples and the strains of lactic acid bacteria used in the present study.

Samples Abbreviation	Lactic Acid Bacteria	Food Source from which Isolated
LPC	*Lactobacillus paracasei* (1) ^b^	Korean traditional rice wine (*makgeolli*)
RTJL3	*Lactobacillus paracasei* (2) ^b^	traditional rice wine (*Myeoncheon Dugyeonju*)
RTCL16	*Lactobacillus sakei* ^b^	traditional fermented barley paste
RTCL79	*Lactobacillus pentosus* ^b^	traditional fermented barley paste
KR10	*Lactobacillus brevis* (1) ^c^	radish kimchi
KR7	*Lactobacillus brevis* (2) ^c^	kimchi
RTJL4	*Lactobacillus hilgardii* ^c^	traditional rice wine (*Myeoncheon Dugyeonju*)
RTCL3	*Pediococcus pentoseceus* ^a^	traditional fermented barley paste
RTCL31	*Pediococcus lolii* ^a^	traditional fermented barley paste
JKA1-6	*Leuconostoc mesenteroides* (1) ^c^	kimchi
JKF2-2	*Leuconostoc mesenteroides* (2) ^c^	radish kimchi
RTCL9	*Weissella cibaria* ^c^	traditional fermented barley paste

^a^ Obligatorily homofermentative. ^b^ Facultatively heterofermentative. ^c^ Obligatorily heterofermentative.
